# From DNA Radiation Damage to Cell Death: Theoretical Approaches

**DOI:** 10.4061/2010/350608

**Published:** 2010-10-05

**Authors:** Francesca Ballarini

**Affiliations:** Department of Nuclear and Theoretical Physics, INFN-Pavia Section, University of Pavia, via Bassi 6, I-27100 Pavia, Italy

## Abstract

Some representative models of radiation-induced cell death, which is a crucial endpoint in radiobiology, were reviewed. The basic assumptions were identified, their consequences on predicted cell survival were analyzed, and the advantages and drawbacks of each approach were outlined. In addition to “historical” approaches such as the Target Theory, the Linear-Quadratic model, the Theory of Dual Radiation Action and Katz' model, the more recent Local Effect Model was discussed, focusing on its application in Carbon-ion hadrontherapy. Furthermore, a mechanistic model developed at the University of Pavia and based on the relationship between cell inactivation and chromosome aberrations was presented, together with recent results; the good agreement between model predictions and literature experimental data on different radiation types (photons, protons, alpha particles, and Carbon ions) supported the idea that asymmetric chromosome aberrations like dicentrics and rings play a fundamental role for cell death. Basing on these results, a reinterpretation of the TDRA was also proposed, identifying the TDRA “sublesions” and “lesions” as clustered DNA double-strand breaks and (lethal) chromosome aberrations, respectively.

## 1. Introduction

Cell death is a crucial issue in radiation-induced biological damage, not only because it is widely considered as a reference endpoint to characterize the action of ionizing radiation in different biological targets, but also because cell killing is the main aim for any tumour therapy. In particular, the dependence of cell-death RBE (Relative Biological Effectiveness) on the particle energy is a fundamental information for Carbon-ion therapy; with increasing depth in tissue, the primary-beam energy decreases, thus implying an increase in the radiation LET (Linear Energy Transfer) and in its biological effectiveness. 

Many experimental data sets on different cell lines cultured *in vitro* and exposed to different radiation fields are available. These data show that while haploid yeasts, some bacteria and spermatogonia are characterized by a purely exponential dose-response curve, which becomes a straight line in the usual semilogarithmic representation, diploid yeasts and almost all mammalian cells follow a “sigmoid” survival curve, which in a semilogarithmic plot is characterized by an initial “shoulder” followed by an almost straight portion. The shoulder is generally ascribed to a multiple-track radiation action whereas the straight portion is thought to be due to the action of single tracks. As a consequence, for high-LET radiation, where the intratrack action predominates, the shoulder tends to disappear and high-LET survival curves can be described by a purely exponential dose dependence. 

To better understand the mechanisms governing radiation-induced cell death and to allow for predictions where the experimental data are not sufficiently reliable (typically at high doses, where the error bars are generally large due to poor statistics), many theoretical models of radiation-induced cell killing have been proposed [1]. These models have in common a few basic assumptions: (1) cell inactivation is the result of a multistep process, where the first step is an energy absorption in one or more intracellular sensitive volumes; (2) energy absorption in the form of ionizations or excitations in the critical volume(s) will lead to molecular lesions in the cell; (3) the processing of such lesions causes the cell to lose its ability to carry out normal DNA replication and cell division. 

In this paper, some of these models will be presented and discussed, focusing on the assumptions adopted by the authors and on possible model advantages and drawbacks. Far from being exhaustive, this overview will first deal with three “historical” approaches: Lea's “Target Theory”, which is one of the earliest interpretive models, the “Linear-Quadratic model”, which overcomes some inconsistencies of the Target Theory with mammalian data and explicitly takes into account DNA damage induction and repair, and the Theory of Dual Radiation Action, which leads as well to a linear-quadratic expression for cell survival but starting from a different background with respect to the LQ model. These three general models will be followed by two approaches (the Katz' model and the more recent Local Effect Model) that are specific for heavy ions; in particular the LEM approach, which allows calculation of heavy-ion cell survival starting from photon experimental data, is applied at GSI in Germany for the biological optimization of Carbon-ion treatment planning. Finally, an original approach developed at the University of Pavia basing on the link between chromosome aberrations and cell death will be presented, together with recent model predictions on the survival of V79 cells exposed to different radiation types. The peculiarity of this approach consists in being mechanistic, since theoretical survival curves are derived from first principles on the biophysical mechanisms underlying DNA damage induction and repair. Importantly, in contrast with many literature mechanistic models including the Linear-Quadratic approach, only two (semi-)free parameters are adopted. Also, the model presented herein works both for low-, intermediate-, and high-LET radiation.

## 2. Lea's Target Theory

Lea's “target theory”, which was first developed in 1946 and published in a refined version in 1955 [2], is one of the earliest interpretive models for radiation-induced cell killing and was developed starting from data on microorganisms and bioactive molecules. According to Lea's model, which is specific for low LET radiation (so that the interaction between distinct events is rare), a cell contains one or more sensitive targets of size *v*, which can receive one or more radiation “hits"; a hit is an “active event" occurring within the volume *v*, that is an energy absorption event able to induce biological damage such as an ionization or an excitation in the target molecule(s) or in water. The hit probability is then *ρ* = *v*/*V*, where *V* is the *total* cellular volume (that is the product between average cell volume and number of cells at risk). If *D* is the total number of active events in the cell population, the probability for a cell to be hit *h* times can be expressed as follows:

(1) p(ρ,h,D)=ρh  (1−ρ  )(D−h)CDh,

where _*D*_ *C*_*h*_ is the binomial coefficient expressing that *h* hits and (*D* − *h*) “misses" can be assigned for *D* active events. Introducing a function *H*(*h*) representing the probability that the cell will survive *h* hits (“hit-survival function"), the survival probability after *h* hits is

(2) P(ρ,h,D)=H(h)p(ρ,h,D)=H(h)ρh(1−ρ  )(D−h)CDh.

Since a cell may survive for *h* = 1,2, 3,…, *D*, the total survival probability for the cell, that is the general survival equation according to Lea's theory, is

(3) $$S\left(\rho ,D\right)=\overset{}{\underset{h}{\sum }}P\left(\rho ,h,D\right).$$

The case that found the widest applicability in radiobiology is the “multitarget-single-hit” (MTSH) version, according to which the cell contains *n *critical targets, each target has the same probability *q *of being hit by radiation, and one hit in a given target is sufficient to inactivate that target but not the entire cell. The probability that a cell will survive with *b* hits is then

(4) P(q,b,n,D)=(1−e−qD)b(e−qD)n−bCbnB(b),

where *B*(*b*), analogous to *H*(*h*), is the hit survival function. In the MTSH case, the following limiting conditions can be assigned to *B*(*b*): (1) if *b* < *n*, *B*(*b*) assumes a value so that *P* = 1; (2) if *b* ≥ *n*, *B*(*b*) = 0 and *P* = 0. This means that for *b* < *n* the cell will survive, whereas for *b* ≥ *n* the cell will die.

Since the *n*th hit assures nonsurvival, the overall survival probability is

(5) $$S\left(q,n,D\right)=1-{\left(1-e^{-qD}\right)}^{n}.$$

It is important to observe that, when ln *S* is plotted versus *D*, except for the special case of *n* = 1 each curve has a “shoulder" that increases in breadth with *n*. Furthermore, for *S* values below about 0.1 each curve becomes a straight line; the extrapolation of this straight line back to the zero-dose ordinate provides the value of *n*, called the “target multiplicity". If the linear portion of the plot is back extrapolated to cross the *S* = 1 ordinate, that intercept is the “quasi threshold dose" *D*_*q*_, which is related to *n* and *D*_0_ (where *D*_0_ = 1/*q* is the dose for 1/*e* survival in the linear portion of the plot) by *D*_*q*_ = *D*_0_ln *n*. Except for *n* = 1, the slope at zero dose will be zero, which is one of the main limitations of the model because it is not consistent with the experimental data. To overcome such limitation, a single-target-single-hit term was included, leading to

(6) $$S\left(p,q,n,D\right)=e^{-pD}\left[1-\;{\left(1-e^{-qD}\right)}^{n}\right],$$

where *p* is the inactivation coefficient for the portion of cell killing that is assumed to arise from single hits, whereas *q* is the inactivation coefficient for the “usual" MTSH model. Although this equation describes fairly well the behaviour of most mammalian cells, however, it still predicts that the slope of the linear portion of the plot remains constant with increasing dose whereas a more frequent experimental observation is a constantly increasing slope. This led the investigators to try alternative approaches, also basing on the fact that starting from the 1960s several investigators reported that their data on mammalian cell lines were better described by functions in which the dose appeared both to the first and to the second power.

## 3. The Molecular (or Linear-Quadratic) Model by Chadwick and Leenhouts

The target theory makes no assumption about the induction and repair of the initial DNA damage, which is now known to play a fundamental role for radiation-induced clonogenic death. A number of alternative approaches to Lea's theory have been developed to respond to such objection, and several of these approaches are of a linear-quadratic form. In particular Chadwick and Leenhouts in 1981 [3] developed what they called the “molecular model", which has come to be widely known as the “linear-quadratic" (LQ) model. According to the LQ model, the cell contains certain critical molecules, assumed to be double-stranded DNA, the integrity of which is essential for clonogenic survival; the critical damage is assumed to be a DNA double-strand break (DSB). Ionizing radiation can induce the rupture of DNA molecular bonds (“lesions") that, under certain conditions, are repaired; varying degrees of repair imply different radiobiological effects. If *N*_0_ is the number of DNA molecular bonds available for rupture in the target cell, *N* is the number of these bonds that remain intact after a dose *D*, and *K* is the rupture probability of a single bond per unit dose, then

(7) $$-\frac{dN}{dD}=KN,\;N=N_{0}e^{-KD}.$$

The number of *effective* broken bonds is therefore

(8) $$N_{0}-N=fN_{0}\left(1-e^{-KD}\right),$$

where *f* is the fraction of broken bonds that are *not *repaired. 

According to Chadwick and Leenhouts, the double helix can undergo a DSB as the result of two different mechanisms: (i) both DNA strands are broken by the same radiation track (or “event"); (ii) each strand is broken independently, but the breaks are close enough in time and space to lead to a DSB. Let ∆ be the fraction of dose acting through mechanism (i), and (1 − ∆) the fraction of dose acting through mechanisms (ii). The number of *unrepaired* DSBs per cell produced by mechanism (ii) is then

(9) $$Q_{ii}=Ef_{0}q_{1}q_{2},$$

where *E* is the “effectiveness factor", that is the likelihood for a DSB to occur from two SSBs associated in time and space, *f*_0_ is the fraction of unrepaired DSBs, and *q*_1_ and *q*_2_ are the number of broken bonds on strands 1 and 2, respectively. Therefore, *q*_1_ = *f*_1_ *n*_1_(1 − *e* ^−*k*(1−  ∆)*D*^) and *q*_2_ = *f*_2_ *n*_2_(1 − *e* ^−*k*(1−∆)*D*^), where *n*_1_ and *n*_2_ are the number of critical bonds on strands 1 and 2, respectively, *f*_1_ and *f*_2_ are the fractions of unrestored bonds in strands 1 and 2, and *k* is the probability of bond rupture per bond and per unit dose. Adopting a similar notation, the number of *unrepaired* DSBs induced via mechanism (i) is

(10) $$Q_{i}=n_{0}f_{0}\left[1-\exp\;\left(-k_{0}∆D\right)\right],$$

where *n*_0_ is the number of DNA sites that can sustain a DSB and *k*_0_ is the hit probability constant.

The average number of DSBs per cell is therefore *Q*_*i*_ + *Q*_*i* *i*_, and the average number of *lethal* DSBs per cell is

(11) $$\begin{array}{c} Q=p\left(Q_{i}+Q_{ii}\right), \end{array}$$

where *p* is the assumed proportionality constant between the DSB yield and cell death. Lumping constants,

(12) $$\begin{array}{c} Q=p\left[\;\chi \left(1-e^{-k0∆D}\right)+\varphi {\left(1-e^{-k(1-∆)D}\right)}^{2}\right]. \end{array}$$

Since according to Poisson-type cell killing the probability of cell survival *S* is given by the probability of having 0 lethal lesions, then *S* = *e* ^−*Q*^. Assuming that *k* and *k*_0_ are quite small, one gets the familiar linear-quadratic relationship as follows:

(13) $$\begin{array}{c} S=\exp\;\left(-\alpha D-\beta D^{2}\right), \end{array}$$

where *α* = (*f*_0_,*n*_0_,*k*_0_,∆) and *β* = (*f*_0_,*E*,*n*_1_,*n*_2_,*f*_1_,*f*_2_,*k* ^2^,(1−∆)^2^). This model represents an attempt to bridge the gap between physics, that is energy deposition by radiation, and biology, that is DNA repair or lack of repair, although the fundamental assumptions are not widely accepted; in particular the hypothesis that the yield of DSBs is proportional to the yield of lethal lesions is not consistent with most experimental data, which in general show that DSBs tend to increase linearly with dose whereas lethal lesions increase with dose in a linear-quadratic fashion. However, the LQ model is widely used in radiobiology since it fits mammalian cell survival data pretty well, overcoming not only the problem of zero slope at zero dose, but also the problem of constant slope at high doses.

## 4. The Theory of Dual Radiation Action by Kellerer and Rossi

The Theory of Dual Radiation Action (TDRA) was proposed by Kellerer and Rossi in 1972 [4], partly to explain the observed increase of neutron RBE at low doses, and partly to incorporate the ideas of microdosimetry. This theory is based on the following assumptions: (1) ionizing radiation induces cellular “sublesions", which are proportional to the radiation dose; (2) the interaction between two sublesions can produce a “lesion", which has a certain fixed probability to lead to cell death. Such interaction is possible only within a “sensitive site"; the interaction probability is 1 for distances smaller than the sensitive site linear dimensions, which according to TDRA are of the order of the micrometer whereas it is 0 for larger distances.

According to the TDRA, the average number of lesions after a dose *D* can be expressed as follows:

(14) E(D)=∫E(z)f(z,D)dz,

where *f*(*z*, *D*)*d* *z* is the probability that, for a dose *D*, the specific energy (that is the energy imparted per event and per unit mass) is between *z* and *z* + *d* *z*, and *E*(*z*) is the average number of lesions within a sensitive site. Since *z* is a measure of the number of sublesions and since sublesions interact in pairs, *E*(*z*) = *k* *z* ^2^ where *k* is a biological property of the system. Therefore,

(15) $$E\left(D\right)=\int {kz}^{2}f\left(z,D\right)dz=k\langle z^{2}\rangle .$$

Basing on their microdosimetry experiments, Kellerer and Rossi rewrote this equation by means of the specific-energy spectra, that is,

(16) $$\langle z^{2}\rangle =\left(\frac{\int z_{1}^{2}f\left(z_{1}\right){dz}_{1}}{\int z_{1}f\left(z_{1}\right){dz}_{1}}\right)D+D^{2},$$

where *f*(*z*_1_) is the distribution of single-event specific energies. *E*(*D*) can therefore be expressed as follows:

(17) $$E\left(D\right)=k\left(\zeta D+D^{2}\right),$$

and the survival probability gets the following linear-quadratic form:

(18) $$S=\exp\;\left[-k\left(\zeta D+D^{2}\right)\right].$$

This theory has been criticized by various authors in the 1980s considering the LQ approach and results of “event-by-event” radiation track structure studies, according to which the interaction between distinct events occurs at the nm level whereas the TDRA sensitive sites have linear dimensions of the order of the micrometer. 

However, an alternative interpretation may be the following: on the basis of the observed relationship between some chromosome aberration types and cell death (see below), sublesions can be thought as DSBs whereas lesions can be thought as lethal chromosome aberrations such as dicentrics and rings; this way a sensitive site of the order of the micrometer becomes consistent with the data, since chromosome aberrations are produced by pairwise interaction of DSBs, and such interaction occurs at the level of interphase chromosome domains, which are known to have linear dimensions of the order of the micrometer.

## 5. Katz' Amorphous Track Structure Model

In the late 1960s [5], Katz proposed an approach for heavy-ion cell killing (called “amorphous track structure model”) focused on the role of radiation track structure. According to this approach, the critical target in a mammalian cell is assumed to be a “substructure” of the cell nucleus with typical linear dimensions of the order of 1 *μ*m; the nucleus contains several of these substructures, “like beans in a bag”. The average energy deposition in a given target volume is assumed to be sufficient to determine the biological response, regardless of the target fine structure at lower scales. To represent the photon dose response, Katz adopted the MTSH version of the target theory, according to which the cell survival probability after a dose *D* can be expressed as follows:

(19) $$S_{\gamma }\left(D\right)=1-{\left[1-\exp\;\left(-\frac{D}{D_{0}}\right)\right]}^{n},$$

where *n* is the number of targets and *D*_0_ is the dose for 1/*e* survival in the linear portion of the plot. Since for heavy ions straight exponential curves are observed, the cell survival probability for heavy ions can be expressed as follows:

(20) $$S_{i}=e^{-\sigma F},$$

where *F* is the particle fluence and *σ* is the “inactivation cross section”. 

According to Katz' approach, the transition from the shouldered curves observed at low LET and the high-LET exponential curves is modelled by attributing the dose to two different inactivation modes, that is the “*γ*-kill” and the “ion-kill”. To determine the relative fractions of energy deposited according to the “*γ*-kill” or the “ion-kill” mode, the inactivation cross section representing the ion-kill contribution is calculated by

(21) $$\sigma =2\pi \overset{}{\underset{}{\int }}{\left[1-\exp\;\left(-\frac{D\left(r\right)}{D_{0}}\right)\right]}^{n}rdr,$$

where *D(r)* is the average dose over a (cylindrical) target with typical size of 1 *μ*m. The relative dose contributions for the ion-kill and the “*γ*-kill” mode are then

(22) $$D_{i}=\left(\frac{\sigma }{\sigma_{0}}\right)D,\;\;D_{\gamma }=D-D_{i}=\left(\frac{1-\sigma }{\sigma_{0}}\right)D,$$

where *σ*_0_ is a “saturation cross section” that is essentially defined by the projected area of all subtargets in the cell nucleus. The total survival probability is then

(23) $$S=S_{i}\cdot S_{\gamma }=e^{-\sigma F}\left[1-{\left(1-e^{-D_{\gamma }/D_{0}\;}\right)}^{n}\right].$$

Although in Katz' model the details at the nm level are not taken into account, it is interesting to note that the linear dimensions of its “critical targets” (~1 *μ*m) are of the same order of the dimensions of mammalian cell interphase chromosomes.

## 6. The Local Effect Model (LEM)

A more recent approach, developed and used at GSI for the biological optimization of Carbon-ion treatment planning, is the “Local Effect Model” (LEM) [6]; this model is based on the assumption that the *local *biological effect, that is the damage in a small subvolume (nm) of the cell nucleus, is solely determined by the expectation value of energy deposition in that subvolume, independent of the radiation type. Given a biological target, this implies that differences in the biological action of charged particle beams should be attributed to the different pattern of energy deposition by heavy charged particles with respect to photons, that is radiation track structure at the nm scale. Furthermore, for a given radiation type, differences in the photon response for different biological targets should lead to differences in the corresponding RBE values. For photons, the volumetric density of *lethal* events per cell can be written as follows:

(24) $$\nu_{X}\left(D\right)=\frac{\langle N_{X}\left(D\right)\rangle }{V_{n}}=\frac{-\ln\;S_{X}\left(D\right)}{V_{n}},$$

where 〈*N*_*X*_(*D*)〉 is the average number of lethal events per cell induced by a dose *D* of photons, *V*_*n*_ is the cell nucleus volume, and *S*_*X*_(*D*) is the fraction of surviving cells for photons. Given the complete local dose distribution *d*(*x*, *y*, *z*) for ion irradiation, the average number of lethal events per cell by heavy ions can be obtained integrating the local-event density *v*_ion_(*d*(*x*, *y*, *z*)) as follows:

(25) $$\langle N_{\text{ion}}\rangle \;=\int \nu_{\text{ion}}\left(d\left(x,y,z\right)\right){dV}_{n}.\;$$

Since according to the fundamental assumption of LEM *ν*_ion_(*d*) = *ν*_*X*_(*d*), the average number of lethal events per cell for heavy ions can be written as follows:

(26) $$\langle N_{\text{ion}}\rangle =\int \frac{-\ln\;S_{X}\left(d\left(x,y,z\right)\right)}{V_{n}}{dV}_{n}.$$

The integration volume for (26) is given by the volume of the cell nucleus, which is represented as a cylinder with axis parallel to the particle trajectory. Since the integrand is fully determined by the photon biological response, cell killing by heavy ions can be calculated starting from photon experimental data, being the heavy-ion effect “hidden” in the inhomogeneous distribution of local dose *d*(*x*, *y*, *z*). Equation (26), which is the most general formulation of LEM, does not rely on any particular representation of the photon dose response curve and can be applied even if only numerical values of *S*_X_ are available. However, for practical reasons, the authors described the photon response by a (modified) linear-quadratic approach, that is,

(27) $$\langle N_{X}\left(D\right)\rangle =-{\ln\;S}_{X}\left(D\right)=\alpha_{X}D+\beta_{X}D^{2}.$$

To take into account that for many biological targets a transition from the shouldered to an exponential shape of the survival curve is observed at high doses, a modified version of the linear-quadratic approach was introduced; according to this version, such transition is described by a parameter *D*_*t*_, representing the transition dose to an exponential shape with slope *s*_max_ = *α*_*X*_ + 2*β*_*X*_ *D*_*t*_. The dose response is then given by

(28) $$-{\ln\;S}_{X}\left(D\right)=\{\begin{array}{cc} \alpha_{X}D+\beta_{X}D^{2}, & D\leq D_{t},\\ \alpha_{X}D_{t}+\beta_{X}D_{t}^{2}+s_{\max\;}\left(D-D_{t}\right), & D>D_{t}. \end{array}$$

Since for most mammalian cell lines survival curves can be reliably measured only down to 10^−3^, in general *D*_*t*_ cannot be directly derived from experimental data, and thus it represents a semifree model parameter; in general, values in the range 15–30 Gy allow consistent descriptions of the data. To perform the numerical integration given in (26) for a random distribution of particle traversals, a grid has to be used to cope with the rapid variation of the radial dose profile, according to the 1/*r* ^2^ distribution. Since this leads to computing times that are unacceptable for treatment planning, approximations have been developed to estimate the *β* parameter. The current version of the model, which recently has been refined taking into account free-radical diffusion, DNA strand-break clustering, and an extension of the inner part of the particle track [7], led to a good agreement with Carbon-ion survival data for different particle energies and different cell lines. 

The LEM approach has been implemented in the TRiP treatment planning procedure for the Carbon-ion therapy project at GSI. According to LEM, the biological characteristics of the various target tissues are essentially determined by the *α*/*β* ratio for conventional photon irradiation and by *D*_*t*_; values of *α*/*β* =  10 Gy are frequently reported for early-responding normal and tumoral tissues whereas *α*/*β* =  3 Gy is reported for late-responding normal tissues. However, the authors themselves emphasize that the photon parameters specific for the considered tissue and the considered endpoint should be used to estimate the RBE. Ideally, *α*/*β* values from clinical data would be appropriate; if these are not available, the corresponding data from *in vivo* studies should be used, and if those are not available*, in vitro* experiments may help.

## 7. A Mechanistic Approach Starting from Chromosome Aberrations

At the University of Pavia, a mechanistic model and a Monte Carlo code originally developed for predicting chromosome aberration induction have been recently extended to simulate cell death, starting from the experimentally-observed relationship between some chromosome aberration types (dicentrics, rings, and deletions) and clonogenic inactivation (e.g., [17]). 

The model/code for radiation-induced chromosome aberrations, which was initiated more than ten years ago [8], relies on the following basic assumptions: (1) chromosome aberrations arise from DNA breaks that are clustered at the nm level (Cluster Lesions, CLs), each lesion giving rise to two independent chromosome free ends; (2) only pairs of chromosome free ends initially induced within a threshold distance *d* can join and thus produce exchange-type aberrations. These assumptions start from the evidence that, on average, 1 Gy of (low-LET) radiation induces about 40 DNA double-strand breaks (DSBs) per cell, but less than 1 chromosome aberration per cell; it is therefore very likely that, among the many initially-induced breaks of the double helix, only those that are severe enough (like those that are clustered at the nm level, assumption 1) and close enough (assumption 2) are involved in the formation of chromosome aberrations, typically *via* the well-known mechanism of nonhomologous end joining (NHEJ). The choice of a step-like function with threshold *d* for the free-end rejoining probability reflects the evidence that DNA repair takes place mainly within the channels separating the various interphase chromosome domains; this evidence was quantitatively “translated” in the simulation code assuming that two chromosome free ends induced with initial distance smaller than a threshold value *d* will join with 100% probability whereas two free ends with larger initial distance will never join. The value of *d* reflects the average linear dimensions of interphase chromosome territories, that is of the order of ~1 *μ*m for mammalian cells.

 The current version of the code can deal either with spherical cell nuclei or with cylindrical nuclei, with dimensions that can be chosen by the user from the input file. The various interphase chromosome territories are modelled as (irregular) intranuclear regions consisting of the union of small adjacent cubic voxels of 0.2 *μ*m side; the volume of each territory, that is the number of voxels constituting that territory, is proportional to the chromosome DNA content. Repetition of chromosome territory construction with different chromosome positions within the cell nucleus provides different interphase nucleus configurations. 

The yield of radiation-induced Cluster Lesions (i.e., average number of CLs per Gy and per cell) is the starting point for the simulation of dose-response curves. In terms of biophysical mechanisms, CLs represent those initial DNA breaks that, being clustered and thus severe, can “evolve” into chromosome aberrations; therefore, the yield of CLs primarily depends on radiation quality (that is radiation type and energy), but it can also be modulated by the repair ability of the specific cell line under consideration. In previous works on chromosome aberration induction in lymphocytes exposed to protons or alpha particles, the CLs yields have been taken from “event-by-event” radiation track-structure simulations in which a CL has been defined as “at least two SSBs on each DNA strand within 30 base pairs” [8–11]. In more recent works [12–14] and herein, to take into account not only the radiation quality but also the specific cell response, such yields were left as a semifree parameter; here “semifree” means that only values ranging between ~1 CLs Gy^−1^  cell^−1^ (low-LET radiation) and ~10 CLs Gy^−1^  cell^−1^ (high LET) are considered as acceptable. 

 For a given irradiated cell, for sparsely-ionizing radiation like X- and gamma-rays an actual number of CLs is extracted from a Poisson distribution, and such lesions are then randomly distributed in the nucleus. For light ions like protons and alpha particles, an actual number of particle tracks traversing the nucleus is extracted from a Poisson distribution with average value *n* = *S* *D*/(0.16 L), where *S* is the nucleus cross-sectional area in *μ*m^2^, *D* is the absorbed dose in Gy, L is the radiation LET in keV/*μ*m, and 0.16 is a numerical factor coming from the conversion of eV into Joules. For each nucleus traversal, an actual number of lesions is then extracted from a Poisson distribution with average value *t* · CL/*μ*m, where *t* is the traversal length in *μ*m and CL/*μ*m is the average number of lesions per unit length, which is calculated as CL/*μ*m = CLs Gy^−1^  cell^−1^ 0.16 L V^−1^, being V the cell nucleus volume in *μ*m^3^. For light ions such as protons and alpha particles, the CLs induced by a given particle are randomly distributed along segments representing the primary particle track. The lesions induced by heavy ions like Carbon, which is nowadays of great interest for tumour hadrontherapy, and Iron, interesting for space radiation research, are located partly along a segment representing the core of the primary track, and partly with a “radial shift” with respect to the track core, to reproduce the effects of energetic secondary electrons (“delta rays”). For a given heavy-ion track, the probability of having a lesion at distance *r* from the track core is assumed to be proportional to *r* ^−2^. 

 After assigning the spatial positions of each CL in the cell nucleus, the subsequent simulation steps consist of (1) identification of the chromosome(s) and chromosome arm(s) that have been hit by each CL; (2) pairwise rejoining between chromosome free ends, assuming 100% rejoining probability if the (initial) distance between the two free ends is <*d* and no rejoining if the distance is >*d*; accidental eurejoining, that is rejoining with the original partner, is allowed; (3) aberration scoring; (4) repetition for a statistically significant number of irradiated cells; (5) repetition for different dose values to obtain a dose-response curve for the main aberration types (dicentrics, translocations, rings, deletions, and more than 40 different complex exchanges), directly comparable with experimental data. Specific background (i.e., prior to irradiation) yields for different aberration types can be included by the user (typically, 0.001 whole-genome dicentrics/cell and 0.005 whole-genome translocations/cell). Both Giemsa staining and whole-chromosome FISH painting can be simulated, and the scoring of chromosome fragments smaller than a threshold value can be “switched off” by the user, since these fragments can hardly be detected experimentally when chromatin is in its condensed state; a threshold value of 10 Mbp (FISH) or 15 Mbp (Giemsa) has been used until now. 

Up to now, the model has been validated for the induction of the main types of chromosome aberrations in lymphocytes exposed to X- and *γ*-rays [10], protons and alpha particles [9], and Carbon ions and Iron ions [11]. The agreement between model predictions and literature experimental data supports the model assumptions on the mechanisms governing chromosome aberration induction, including the fundamental role of DNA damage clustering at the nm scale and the step-like distance dependence at the *μ*m scale for the rejoining probability between two (clustered) DNA lesions. Furthermore, the model has been applied to predict the induction of Chronic Myeloid Leukaemia following acute exposure to gamma rays [15] and the induction of chromosome aberrations in astronauts exposed to space radiation [16]. 

The model/code described above has recently been extended to simulate radiation-induced cell death, starting from the experimentally observed one-to-one relationship between the average number of “lethal aberrations” (that is Giemsa-stained dicentrics, rings, and deletions) per cell and −ln *S*, where *S* is the fraction of surviving cells [17]. In this extended version, the fraction of surviving cells after a dose *D* is then calculated as S(*D*) = e^−*L* *A*(*D*)^, where *L* *A*(*D*) is the (simulated) average number of lethal aberrations per cell. While the experimental study by Cornforth and Bedford concerns AG1522 cells exposed to X-rays (and subject to delayed plating to allow for potentially-lethal damage repair), the theoretical approach reported herein was applied also to protons, *α*-particles, and Carbon ions. The first step of the work consisted of reproducing the experimental outcomes on X-irradiated AG1522 cells; the very good agreement between model predictions and experimental data confirmed the important role of lethal aberrations for radiation-induced cell death [13]. Subsequently, the approach has been extended to V79 cells exposed to X- or gamma-rays, 0.76 MeV protons (LET: ~30.5 keV/*μ*m), and 3.2 MeV *α*-particles (LET: ~120 keV/*μ*m). Good agreement with literature experimental data [18–20] was found for all the considered exposure scenarios, indicating that the relationship between lethal aberrations and cell death observed by Cornforth and Bedford can hold not only for AG1522 cells exposed to X-rays but also for other cell types and other radiation types. Very recently, the approach was applied also to 11.0 MeV/u Carbon ions (LET: 153.5 keV/*μ*m). 

**Figure 1:**
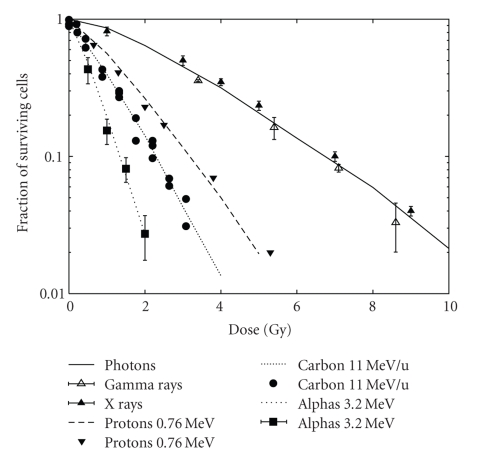
From top to bottom: survival of V79 cells exposed to photons, 0.76 MeV protons, 11.0 MeV/u Carbon ions, and 3.2 MeV alpha particles; the lines are model predictions, the points are experimental data taken from [18–21]. The cell nuclei were modelled as right cylinders with height 6 *μ*m and radius 6 *μ*m (radius 5 *μ*m for the Carbon data, basing on personal communication).

The results described above are reported in Figure 1, which shows cell survival curves obtained for V79 cells exposed to photons, 0.76 MeV protons, 3.2 MeV *α*-particles, and 11.0 MeV/u Carbon ions. The lines are model predictions whereas the points are the corresponding literature data chosen for comparison, that is V79 cells exposed to X-rays [18], *γ*-rays [19], 0.76 MeV protons [20], 3.2 MeV *α*-particles [19], and 11.0 MeV/u Carbon ions [21].

## 8. Conclusions

Some representative theoretical models of radiation-induced cell inactivation were presented and discussed, outlining the main biophysical assumptions adopted by the various authors and analyzing the consequences of such assumptions in terms of cell survival predictions. More specifically Lea's target theory, which was developed before the discovery of the DNA double helix for microorganisms exposed to low-LET radiation, in its MTSH version assumes that there exist **n** critical targets in the cell, and that the *n*th radiation hit ensures nonsurvival; therefore, the predicted survival curve in the usual semilog scale is characterized by an initial shoulder (with zero slope at zero dose) followed by a straight portion. While the MTSH model fits quite well bacteria survival data, most mammalian cell data follow a linear-quadratic behaviour, with negative slope at zero dose and increasing slope at high doses. 

Both the Molecular Model and the Theory of Dual Radiation Action lead to a linear-quadratic survival curve, though following different approaches. The Molecular Model considers the DNA double helix as the critical target and takes into account the biophysical mechanisms of DNA damage induction and repair, assuming that the critical damage is a DSB (which can be induced either by a single-track action or by a two-track action) and that the yield of DSBs is proportional to cell inactivation. On the contrary, the TDRA, without specifying the critical target(s) nor the critical damage(s), assumes that radiation induces cellular “sublesions” that interact in pairs to produce “lesions”, which in turn lead to cell death with a certain fixed probability; such interaction can take place only within “sensitive sites” of the order of ***μ*** m. While these three models focus on the general mechanisms of cell death induced by low-LET radiation, Katz' approach and the LEM model are specific for heavy-ion applications. In particular the LEM model, which describes heavy-ion cell death starting from photon experimental data, is used at GSI in Germany for the biological optimization of Carbon-ion treatment planning. 

After reviewing the literature models mentioned above, an approach developed at the University of Pavia was presented together with recent results. The peculiarity of this approach consists in the fact that on one side it is mechanistic (that is the model predictions are derived from a few basic assumptions on the biophysical mechanisms underlying radiation-induced cell death), but at the same time the number of free parameters is kept at a minimum, since only two semifree parameters are adopted: the yield of DNA “Cluster Lesions” and the value of the threshold distance *d* for chromosome free-end rejoining. More specifically, the model may be taken as a mechanistic reinterpretation of the TDRA, since the TDRA “sublesions” and “lesions” can be (re-)interpreted as DNA “Cluster Lesions” and (lethal) chromosome aberrations, respectively; CLs, which increase with dose linearly, interact in pairs to produce chromosome aberrations, which increase with dose in a linear-quadratic fashion; such interaction occurs at the level of interphase chromosome domains, which have linear dimensions of the order of the micrometer like the TDRA sensitive sites. 

The good agreement between model predictions and literature experimental data on low-, intermediate-, and high-LET radiation (photons, protons, alpha particles, and Carbon ions) supported the idea that asymmetric chromosome aberrations, such as dicentrics and rings, play a fundamental role in the mechanisms governing radiation-induced cell death. Furthermore, the model may be considered as a predictive tool for potential applications in radiotherapy including therapy with Carbon ions, which is now adopted in various centres worldwide, including the CNAO centre in Pavia.
